# Assessing Repeated Oxalic Acid Vaporization in Honey Bee (Hymenoptera: Apidae) Colonies for Control of the Ectoparasitic Mite *Varroa destructor*

**DOI:** 10.1093/jisesa/ieab089

**Published:** 2022-02-07

**Authors:** Jennifer A Berry, Lewis J Bartlett, Selina Bruckner, Christian Baker, S Kris Braman, Keith S Delaplane, Geoffrey R Williams

**Affiliations:** 1 Department of Entomology, University of Georgia, Athens, GA 30602, USA; 2 Center for the Ecology of Infectious Diseases, University of Georgia, Athens, GA 30602, USA; 3 Entomology & Plant Pathology, Auburn University, Auburn, AL 36849, USA

**Keywords:** honey bee, varroa, oxalic acid, control, IPM

## Abstract

The American beekeeping industry continually experiences colony mortality with annual losses as high as 43%. A leading cause of this is the exotic, ectoparasitic mite, *Varroa destructor* Anderson & Trueman (Mesostigmata: Varroidae). Integrated Pest Management (IPM) options are used to keep mite populations from reaching lethal levels, however, due to resistance and/or the lack of suitable treatment options, novel controls for reducing mites are warranted. Oxalic acid for controlling *V. destructor* has become a popular treatment regimen among commercial and backyard beekeepers. Applying vaporized oxalic acid inside a honey bee hive is a legal application method in the U.S., and results in the death of exposed mites. However, if mites are in the reproductive stage and therefore under the protective wax capping, oxalic acid is ineffective. One popular method of applying oxalic is vaporizing multiple times over several weeks to try and circumvent the problem of mites hiding in brood cells. By comparing against control colonies, we tested oxalic acid vaporization in colonies treated with seven applications separated by 5 d (35 d total). We tested in apiaries in Georgia and Alabama during 2019 and 2020, totaling 99 colonies. We found that adult honey bees Linnaeus (Hymenoptera: Apidae), and developing brood experienced no adverse impacts from the oxalic vaporization regime. However, we did not find evidence that frequent periodic application of oxalic during brood-rearing periods is capable of bringing *V. destructor* populations below treatment thresholds.

The western honey bee, (*Apis mellifera* L.), is a ubiquitously used insect pollinator of many agricultural crops around the world (Allsopp et al. 2008, vanEngelsdorp and Meizner 2010), and the economic services provided by these managed bees has become increasingly important as world population expands (Degrandi-Hoffman et al. 2019). However, in recent years, populations of *A. mellifera* have seen a gradual, yet steady decline (Potts et al. 2010, Spivak et al. 2011, Kulhanek et al. 2017, vanEngelsdorp et al. 2017) with the American beekeeping industry experiencing annual losses of 43.7% (Bruckner et al. 2020). There are a number of drivers involved in colony loss, with the ectoparasitic mite *Varroa destructor* Anderson & Trueman (Mesostigmata: Varroidae) among the most important (Guzmán-Novoa et al. 2010, Le Conte et al. 2010, Rosenkranz et al. 2010).

To date, there are three synthetic acaricides (amitraz, coumaphos, and fluvalinate) approved for use against *V. destructor* in the U.S. (US EPA, 2016), but due to sub-lethal effects on honey bees along with rapidly evolving resistance in *V. destructor* (Elzen et al. 2000, Mathieu and Faucon, 2000, Thompson et al. 2002, Rodríguez-Dehaibes, 2005, Sammataro et al. 2005, Berry et al. 2013, Rinkevich, 2020), there is a need for additional efficacious active ingredients. Because of this, beekeepers have employed integrated pest management (IPM) techniques instead of relying on a single method of control. Beekeepers embracing IPM use a variety of approaches to try and keep colonies from succumbing to the detrimental effects caused by *V. destructor* (Delaplane et al. 2005). However, adequate control of this pest remains a serious challenge for many US beekeepers and chemical acaricides are still necessary as part of IPM frameworks in this system.

Non-synthetic compounds such as formic acid and thymol are effective at controlling *V. destructor*; however, their effectiveness is dependent on ambient conditions. For example, they are not effective when temperatures are too low and may kill adult and developing bees when temperatures are too high (US EPA 2019, US EPA, 2020). Another widely adopted natural compound is crystalline oxalic acid (OA) dihydrate. This organic acid naturally occurs in nectar, has putatively low likelihood of inducing *V. destructor* resistance on account of no resistance yet being observed in treated populations compared to naïve ones despite years of continuous use (Maggi et al. 2017), and has high efficacy against *V. destructor* (Bogdanov et al. 2002, Rademacher and Harz, 2006, Al Toufailia et al. 2015, Adjlane et al. 2016) in certain circumstances. Widely used for decades in Europe, (Popov et al. 1989), OA has only recently been popularized in the U.S. and wasn’t registered for legal use until 2015 (US EPA 2015). One method for applying OA is to heat the crystals using a vaporizer, creating gaseous OA that permeates the colony (Rademacher and Harz 2006, US EPA 2015). Even though these treatments are highly effective at killing *V. destructor* on contact, OA does not penetrate the wax-capped brood cells where the majority of *V. destructor* reside (Rademacher and Hartz, 2006, Rosenkranz et al. 2010). Therefore, the best time to apply OA and reduce *V. destructor* populations is when colonies are broodless, without developing larvae (Gregorc and Planinc, 2001, Charrière and Imdorf, 2002, Gregorc et al., 2016, Gregorc et al., 2017), rendering all mites phoretic on adult bees and vulnerable to the fumigant (Rademacher and Harz, 2006). However, brood-free intervals are brief or absent altogether in some warm latitudes, raising the need for alternative treatment schedules.

For treating during periods of brood rearing, instructions for one commercial vaporizer, the ProVap 110, calls for four treatments with 5 d between each treatment. The rationale for this 19-day interval being that this schedule exposes an entire cohort of mites bound in worker brood as the mites successively emerge with their parasitized hosts. This multiple treatment regimen has gained popularity in commercial and hobby beekeeping operations. However, the protocol has not been shown effective.

The objective of this study was to test the efficacy of a regimen of repeated OA applications against *V. destructor* during periods of brood rearing. A secondary objective was to determine if these repeated OA applications are measurably detrimental to adult bees and brood (proxies for colony viability). We hypothesized that a repeated OA treatment regimen would have a negative effect on *V. destructor* abundance while having no negative effect on *A. mellifera* colony strength, in agreement with prior demonstrations of its relative safety (Rademacher and Harz 2006).

## Materials and Methods

### Experimental Design

Experimental *A. mellifera* colonies were established in the summer months of 2019 & 2020 and maintained in two deep Langstroth hives on research lands maintained by the University of Georgia (UGA) Bee Lab in Watkinsville, GA and the Auburn University Bee Lab in Auburn, AL. Queens, with no specific genotype, were purchased from a commercial operation in North Georgia. Prior to the beginning of the experiment, colonies were assessed and only those that were healthy with productive queens were included. Colonies were not manipulated to be ‘standardized’ in size or brood area beyond all being maintained with equal hive space, so as to accurately capture the variation in colony metrics observed in real apiaries.

In 2019, 13 experimental colonies were set up at the UGA Bee Lab whereas, in 2020, 56 experimental colonies were set up at the UGA Bee Lab and 30 at the Auburn Bee Lab. All colonies had naturally occurring *V. destructor* mite infestation levels (median field-occurring PMI values at the start of each experiment: Auburn20 = 4.3; UGA19 = 5; UGA20 = 2.2). Colonies were randomly assigned to one of two treatment groups: (1) vaporized with 1 g/super of OA every 5 d for seven applications (=7 treatments spread over 35 d) or (2) an untreated control group. The seven application regimen on days 0, 5, 10, 15, 20, 25, & 30 was chosen in order to capture both worker (21 d) and drone (24 d) developmental times.

### Oxalic Acid Application

OA application was administered to colonies by crystal vaporization according to label instructions of the registered product (US EPA 2015) and the user manual for the ProVap 110 Vaporizer (OxaVap, Manning, SC). Prior to vaporization and to ensure that vaporized gas would not leak from the hives, colony entrances were sealed with blue shop towels or duct tape, and screened bottom boards were sealed using corrugated plastic boards. Powered by a Champion 2,000-watt gasoline generator, the vaporizer device, a Pro VAP 110, was inverted and the chamber bowl heated to 230°C. One gram of solid OA dihydrate crystals per deep brood box was placed into the separated Teflon lid and inserted into the chamber. Turning the device right side up caused the OA crystals to fall into the heated vaporizer chamber thereby generating gaseous OA. The nozzle of the device was inserted into the entrance of each UGA colony or into a predrilled hole in the bottom brood box of each Auburn colony, where it remained for 30 s to ensure that the full dose was vaporized and delivered into the colony. Once completed, the device was removed and shop towels and plastic corrugated boards left in place for an additional 10 min per hive. For the safety of all persons applying the OA, full-face respirators with OV/P100 cartridges were worn.

### *Varroa destructor* Abundance

At the beginning (D0), mid (D21), and end of the experiment (D42), *V. destructor* levels were determined by alcohol washes. For each colony, ~ 300 adult bees were collected from the brood nest (Dietemann et al. 2013) and placed into a Varroa EasyCheck device (Mann Lake, Hackensack, MN) filled with 70% ethanol, which euthanizes adult bees and phoretic *V. destructor*. The container was sealed and shaken for 60 s to dislodge *V. destructor* from the adult bees. The adult bees were removed from the container by lifting out the mesh basket, any dislodged individual *V. destructor* counted, recorded, and discarded. The mesh basket of bees was returned into the container, agitated for an additional 60 s, removed, and *V. destructor* counted, recorded, and discarded. This process was repeated until no *V. destructor* were recorded for two consecutive washes. For the Auburn 2020 experiments, each sample of adult bees was weighed. A subsample of 100 ethanol-drenched adult bees was then counted and weighed as a standard, and from this, the expected number of bees in each sample was estimated (Dietemann et al. 2013). *V. destructors* per bee was calculated by dividing total *V. destructor* count for any given sample by the estimated number of adult bees in that sample. For the UGA 2019 and 2020 experiments, the number of bees in each alcohol sample was counted by hand, giving an exact number of adult bees per sample for calculating *V. destructor* per bee estimate.

### Colony Strength

For the 2020 experiments, colony strength variables were measured for each colony at the beginning and end of each experiment by two independent observers who visually estimated percent area coverage of adult bees, developing bees (capped brood), and honey on every hive frame following Delaplane et al. (2013). The total estimate for adult bee population, capped brood, and capped honey (measured in ‘full frames’) was then calculated for each colony and used in analysis.

### Statistical Analysis

Percent mite intensity (PMI) was calculated by measuring mites-per-bee (divided the number of mites found in a sample by the number of bees in that sample as above) and scaled this up to expected number of mites found on 100 bees from that sample. We then calculated the change in PMI for each colony between the start and end of the experiment (∆PMI) by subtracting the PMI from the pre-treatment sample from the PMI of the post-treatment sample, giving a single ∆PMI for each colony. We calculated similar, if simpler, metrics for change in estimates of capped brood, stored honey, and adult bee population. We avoid confounds of any differences in average sizes or infestation levels between colonies randomly assigned to control or treatment by focusing on these ‘change’ values rather than simply comparing colony end-point measures.

All data manipulation and analyses were undertaken in the programming language R (R Core Team, 2020) version 3.6.3. We made the full analysis available as a repository on GitHub (https://github.com/LBartlett/VRTT-OA-Sublimation.git). We analyzed the data using a generalized linear mixed modeling framework (GLMMs) to account for the crossed or nested structure of repeating the experiments in multiple sites and/or apiaries and across multiple years. We used the ‘afex’ package (Singmann et al. 2020) which wraps around the ‘lme4’ package (Bates et al. 2015) to undertake type-III ANOVAs following a Kenward-Roger approximation (see afex package documentation) on linear mixed models, in order to test for significant effects of treatment on the response variables. Models were visually inspected for suitability of fit by graphically examining the distribution of residuals and residual qq-plots. Where appropriate, we used the ‘emmeans’ package (Lenth, 2020) for estimating approximate effect sizes and confidence intervals for plotting data.

## Results

**Fig. 1.**
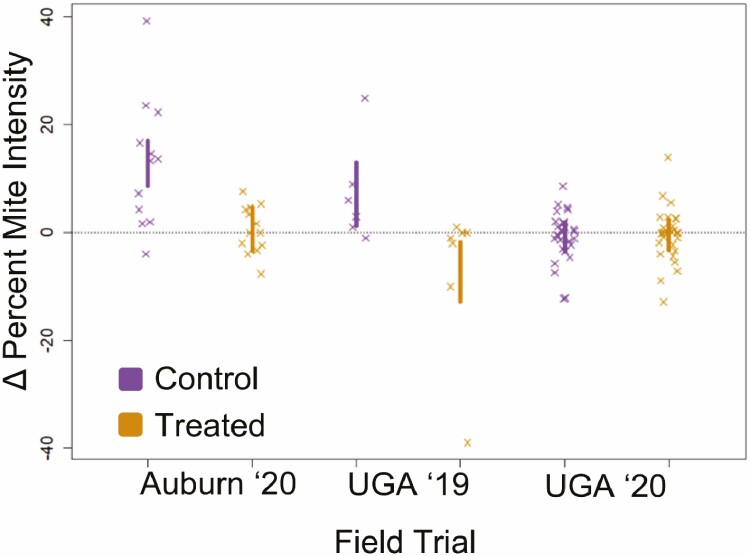
Comparison of ∆PMI (Percent Mite Intensity) by treatment across the locations and years for *Varroa destructor*. Each point represents a single colony, and points are plotted alongside 95% confidence intervals, estimated from naïve linear models (note these naïve regressions are not used for statistical analyses).

For testing the efficacy of repeated 1g oxalic acid sublimation in treating for *Varroa destructor*, we analyzed data from all sites across both years using a linear mixed model with ∆PMI as the response variable, treatment as a fixed effect, year as a standalone random effect and site and apiary as nested random effects to reflect the spatial structuring of the field trials. We found a significant difference in ∆PMI values between control and treated colonies (F_1,88.99_ = 9.16, *p* = 0.003); across the study (35 d) a typical control colony showed an increase in PMI of 4.4 (±2.6 SE), whereas a typical OA treated colony showed a very small decrease in PMI of –0.7 (±2.5SE). As shown in Fig. 1, treated colonies remained at the same PMI after treatment as before (no significant change in PMI, see prior quoted effect size estimates with standard errors spanning zero).

**Fig. 2.**
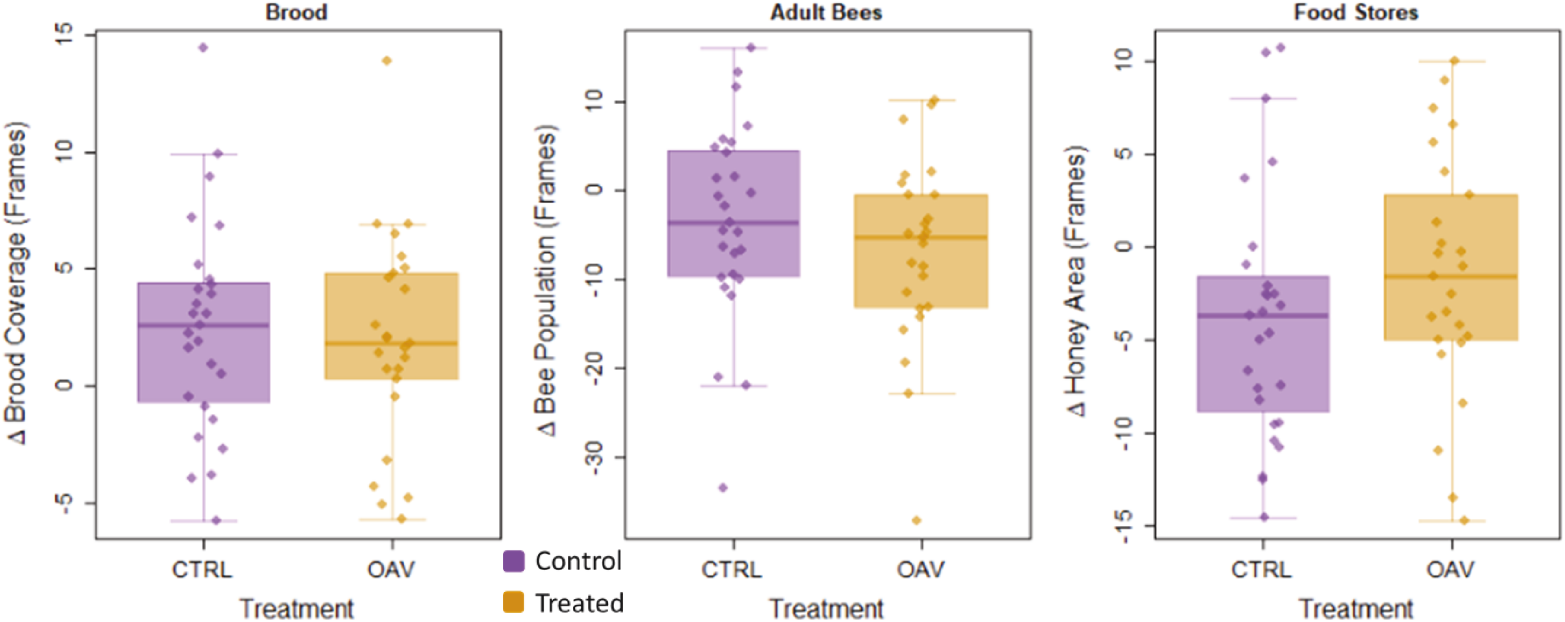
Changes in honey bee (*Apis mellifera*) colony health metrics from the UGA 2020 data grouped by treatment. Brood coverage, adult bees, and honey stores for each colony were estimated (units: standard Langstroth deep frames) before and after the experimental period and the change (delta value) calculated for plotting here and mixed modeling analysis.

As there was no meaningful difference in mite loads between and pre- and post-treatment treated colonies (Fig. 1—UGA 2020), there is no confounding by which *V. destructor* control indirectly improved colony health by masking or compensating for toxic effects of oxalic acid sublimation. For testing the effects of oxalic acid vaporization on overall colony health, we, therefore, present a detailed analysis of the UGA 2020 data (see Fig. 2) as we did not gather detailed colony health data for the UGA 2019 trial data set, and the Auburn 2020 data set was more confounded by a difference in mite control between the two treatments (which will impact colony health, masking possible negative effects of the oxalic acid which compromises assessing safety) compared to the larger UGA 2020 data. Furthermore, this data set was the most replicated experiment, across the three apiaries, and represented the majority of the data. We used a mixed modeling framework as above where response variables were either change in brood area, change in bee population, or change in honey stores, fixed effect was treatment, and random effect was apiary (yard). We found no significant differences in changes in brood (F_1, 52.06_ = 0.39, *p* = 0.534), bees (F_1, 51.23_ = 0.20, *p* = 0.653), or honey stores (F_1, 51.20_ = 2.30, *p* = 0.136) based on treatment.

## Discussion

On average, after 35 d colony *V. destructor* numbers were significantly higher in nontreated controls compared to OA-treated colonies. However, this effect is wholly explained by a small *V. destructor* increase in controls while *V. destructor* levels remained unchanged in OA-treated colonies (Fig. 1). OA did not reduce *V. destructor* numbers; at best, it held them static. This effect is similar to those found by Jack et al. (2020, 2021) in which one and three applications of 1 g of vaporized OA/ super were also ineffective at significantly reducing *V. destructor* infestation levels while brood was present. Additionally, we observed that multiple treatments vaporizing with OA had no significant effects on overall *A. mellifera* adult bees, brood, or stored honey quantity.

Until now, there has only been anecdotal evidence that the recommended vaporizing with OA four times, 5 d apart, results in controlling *V. destructor*. Other studies that have examined the effect of repeated applications of the labeled rate of OA, either by liquid trickling or vaporization, have not shown OA to be effective during the brood-rearing season (Gregorc et al. 2017; Jack et al. 2020, 2021). Studies that examined vaporizing with OA during broodless periods have documented good control of *V. destructor* (Rademacher and Harz, 2006), and for higher doses of 2.25 g permissible outside the U.S. we point to Al Toufailia et al. (2015, 2018) who also demonstrated efficacy of OA in the absence of brood by trickling, spraying and vaporizing.

Correspondingly, our first question was simply whether *V. destructor* infestation levels would be affected by the repeated OA vaporization treatment despite brood being present. Fig. 1 depicts how change in percent mite infestation remained static, hovering around zero (ΔPMI in treated colonies –0.7 (±2.5 SE)). As expected, *V. destructor* levels in control colonies did increase +4.4 (±2.6 SE); hence there was a significant difference between the control colonies and those treated, with OA. However, for a *V. destructor* treatment to be successful, especially when treating colonies that have exceeded the treatment threshold as part of an IPM approach, *V. destructor* infestation must be lowered significantly and not simply remain the same. This raises the question, if colonies are treated with vaporized OA, multiple times, well before *V. destructor* levels reach the treatment threshold, can suitable *V. destructor* control be achieved (explaining anecdotal evidence from beekeepers). It is also a question of whether 1 g OA/super is an effective dose. Al Toufailia et al. (2015) working in the United Kingdom found that vaporizing with 4 times the US-label rate of 1 g per brood box resulted in a 98.2% reduction in *V. destructor* levels. Recently, Jack et al. (2021) demonstrated in Florida that colonies vaporized with 4 g of OA while brood is present had significantly lower infestation levels of *V. destructor* than those vaporized with only 1 g per brood box. Future studies could investigate the efficacy of increased doses of OA on reducing *V. destructor* population levels.

It was already widely known that the most desirable time to treat with OA is when colonies are broodless (Gregorc and Planinc, 2001, Charriere and Imdorf, 2002, Gregorc et al., 2016, Gregorc et al., 2017). Unfortunately, vaporizing with OA does not penetrate the wax capping of the brood cell where *V. destructor* is reproducing (Rademacher and Harz 2006, Rosenkranz et al. 2010) and likely accounts for much of the variance in reported success with OA. Broodless periods naturally occur when the queen seasonally stops laying eggs. Future research should investigate the practicality and effectiveness of forcing or exploiting brood breaks as part of management (Jack et al. 2020). This may be possible by caging the queen, and may be a promising avenue of future research, but this is not always convenient or possible for many beekeepers especially at commercial scales. It may also be possible to exploit brood breaks that occur incidentally as part of normal management such as making splits or requeening.

Our second objective was to determine if multiple applications of OA in a colony have measurable effects on adult bees, brood, and stored honey amounts. The overall higher exposure of OA to the colony could plausibly lead to detrimental effects, especially for developing brood (Higes et al. 1999, Gregorc et al. 2004, Hatjina et al. 2005, Terpin et al. 2019). Our results showed no significant differences in changes in adult bees, brood, or stored honey when colonies were exposed to OA. This supports previous studies with gaseous OA (Al Toufailia et al. 2015; Jack 2020, 2021). Our UGA 2020 data set is well suited for inferring the safety (or lack thereof) as the unchanging *V. destructor* parasitism levels in control and treated populations (Fig. 1) remove potentially confounding effects of OA mitigating effects of the parasite. We consider our results here to be among the strongest demonstrations of the relative safety of OA to *A. mellifera*. Perhaps, future experiments may want to explore the long-term effects and overwintering ability of colonies after being treated with oxalic acid.

Based on our results, we do not recommend employing this method for controlling *V. destructor* when brood are present, especially as a summer or fall treatment option when infestation levels are at or above the treatment threshold in an IPM framework. Even though there was a difference between control and treated groups, colonies vaporized with OA multiple times did not experience a reduction in *V. destructor* infestation levels, and so treatment was ineffective by common standards. It is important for beekeepers to adopt reliable and effective treatment regimes along with realistic, IPM approaches to sustainably reduce infestation levels of *V. destructor*. In 2020 and 2021, two studies which vaporized OA, were successful in significantly reducing *V. destructor* populations. Büchler et al. (2020) vaporized with 2 g of OA while incorporating a brood break and Jack (2021) vaporized with increased amounts of OA (2 g & 4 g) while brood was present. Because of these results, one future study could be to investigate vaporization with increased doses of OA, in conjunction with and without a brood-break.
